# Associations Between Perceived Physical and Mental Fatigability and Life Space Mobility in Older Men: The MrOS Study

**DOI:** 10.1093/geroni/igab046.2161

**Published:** 2021-12-17

**Authors:** Kyle Moored, Andrea Rosso, Theresa Gmelin, Yujia (Susanna) Qiao, Michelle Carlson, Peggy Cawthon, Jane Cauley, Nancy W Glynn

**Affiliations:** 1 University of Pittsburgh, Pittsburgh, Pennsylvania, United States; 2 University of Pittsburgh Graduate School of Public Health, Pittsburgh, Pennsylvania, United States; 4 Johns Hopkins University, Baltimore, Maryland, United States; 5 California Pacific Medical Center, San Francisco, California, United States

## Abstract

Physical performance and fatigue can limit mobility within the larger environment (life-space mobility). It is unknown whether perceived fatigability, fatigue anchored to activity intensity and duration, is independently associated with life-space. We assessed this cross-sectionally in the Osteoporotic Fractures in Men Study (MrOS; N=1,681, Meanage=85±4.1). The Pittsburgh Fatigability Scale (PFS, range: 0-50) measured physical (Mean=16.2±9.5) and mental fatigability (Mean=7.5±8.0). Life Space Assessment scores (range: 0-120, higher=greater life-space) incorporated level, frequency, and assistance used for life-space mobility (Mean=84.3±22.0). Compared to the lowest fatigability strata (Physical: PFS 0-4; Mental: PFS 0-3, modeled separately), men in the two highest physical strata (PFS 20-24: B=-4.10±1.67; PFS≥25: B=-6.23±1.72; p’s≤.05) and men in the three highest mental strata reported significantly lower life-space mobility (PFS 13-15: B=-3.42±1.74; PFS 16-19: B=-5.38±1.83; PFS≥20: B=-7.96±1.66, p’s≤.05), adjusted for physical performance and health covariates. Our results provide evidence linking fatigability and real-world mobility, independent of physical health, in older men.

